# Rapid Intraspecific Diversification of the Alpine Species *Saxifraga sinomontana* (Saxifragaceae) in the Qinghai-Tibetan Plateau and Himalayas

**DOI:** 10.3389/fgene.2018.00381

**Published:** 2018-09-18

**Authors:** Yan Li, Qing-Bo Gao, Zhuo-Ma Gengji, Liu-Kun Jia, Zhi-Hua Wang, Shi-Long Chen

**Affiliations:** ^1^Key Laboratory of Adaptation and Evolution of Plateau Biota, Northwest Institute of Plateau Biology, Chinese Academy of Sciences, Xining, China; ^2^College of Life Sciences, University of Chinese Academy of Sciences, Beijing, China; ^3^Qinghai Provincial Key Laboratory of Crop Molecular Breeding, Xining, China

**Keywords:** intraspecific diversification, *Saxifraga sinomontana*, microrefugia, quaternary glaciations, Qinghai-Tibetan plateau, Himalayas

## Abstract

An increasing number of phylogeographic studies have been conducted for plant species in the Qinghai-Tibetan Plateau (QTP) and its flanking mountains. However, these studies have mainly focused on the determination of glacial refugia and routes of inter-/post-glacial expansions. Rapid intraspecific diversification of plants in this region have not been thoroughly discussed. Herein, we investigate the effects of the Quaternary climate changes on population genetic structure and diversifications of a herbaceous alpine species, *Saxifraga sinomontana*, which may have an evolutionary time scale <5 million years in the QTP and Himalayan regions. Using a total of 350 individuals from 29 populations, we studied the evolutionary history of *S. sinomontana* by analyzing cpDNA *trn*L-*trn*F, *rpl*16 and nrDNA ITS sequences. A total of 89 haplotypes and 158 genotypes were detected for cpDNA and ITS sequences, respectively. Only a few haplotypes/genotypes were widespread, while an extremely large number of haplotypes/genotypes were restricted to single populations, which were scattered throughout the current geographical range of *S. sinomontana*. This suggests the existence of microrefugia of this species during the Quaternary glaciations. In addition, the relationships of the haplotypes/genotypes were almost completely not resolved by phylogenetic reconstruction. Combining characteristics in terms of high haplotype richness, large proportion of private haplotypes, and shallow haplotype divergence, we speculate that recent intraspecific diversification has occurred in *S. sinomontana*. Molecular clock analysis estimated that the onset diversification within *S. sinomontana* to be 1.09 Ma (95% HPD = 0.80–1.45), coinciding with the extensive Quaternary glaciations on the QTP which started ca. 1.17 Ma. The Quaternary climatic oscillations may have triggered rapid intraspecific diversification in this QTP-Himalayan species. However, large niche breadth, as well as introgression/hybridization between the studied species and its closely related sympatric saxifrages, may also played a role to some extent on the current genetic structure of *S. sinomontana*, which need to be further studied.

## Introduction

Evolutionary diversifications are usually considered as one of the main mechanisms that accumulate high levels of plant biodiversity in mountainous regions (Hughes and Eastwood, 2006; Pennington et al., 2010; Hughes and Atchison, 2015). This is the case for alpine plants in the Qinghai-Tibetan Plateau (QTP) and its surrounding Himalayas and Hengduan Mountains region (HHM), from where a large number of rapid diversifications have been recorded (see Wen et al., 2014 and references therein). The driving factors that have triggered such diversifications could be extrinsic (e.g., orogeny and climate change) (Hoorn et al., 2010), intrinsic (e.g., innovation of novel traits, polyploidization, hybridization, and niche shifts), or a combination (Ebersbach et al., 2017b). Among these factors, orogenic events are proposed to have played a disproportionate contribution to plant diversifications in the QTP and HHM at generic, even higher taxonomic levels (e.g., Liu et al., 2006; Wang Y.-J. et al., 2009; Zhang et al., 2012, 2014; Favre et al., 2015, 2016; Ebersbach et al., 2017a,b; Xing and Ree, 2017). In contrast to deeply divergent lineages in which diversifications were mainly triggered by ancient orogeny, intraspecific differentiations of recently divergent species could be shaped by extrinsic events at a small timescale, such as the Quaternary glaciations, which started ca. 1.17 million years ago (Ma; Zheng et al., 2002). Repeated environment alternations between arid/cold glacial and humid/warm inter-glacial episodes may have caused habitat fragmentation which promoted isolation of populations, and thereby facilitated intraspecific differentiation (Hickerson et al., 2010; Gao et al., 2012, 2016; Jia et al., 2012). Due to extremely complex topolography (Favre et al., 2015), extensive width of ecological niches (Ni and Herzschuh, 2011), lack of unified glaciers (Zheng et al., 2002), as well as effects of monsoon systems (Zhang et al., 2000), the evolutionary histories of plants in the QTP and HHM during the Quaternary glaciations seem to be more complicated than in North America and Europe (see Qiu et al., 2011 and references therein). Species that have different distribution ranges, population sizes, mating systems, life forms, and bio-characteristics may have experienced distinctive patterns of divergence and evolutionary history during glaciations (Shahzad et al., 2017). The accumulation of phylogeographic data for as many species as possible can provide a more complete picture of the intraspecific evolutionary history of plants in the QTP and HHM.

Encompassing ca. 500 species in at least 13 sections (Pan et al., 2001; Gao et al., 2015, 2017; Tkach et al., 2015; Ebersbach et al., 2017b), *Saxifraga* L. is the largest genus of Saxifragaceae s.str. (Soltis, 2007). Its distribution is primarily concentrated in the mountainous and arctic regions of the Northern Hemisphere. This species-rich genus has widely been employed in the fields of systematics and phylogeography to reveal patterns and processes of plant diversification in arctic and alpine regions (e.g., Conti et al., 1999; Vargas, 2000, 2001; Abbott and Comes, 2003; Oliver et al., 2006; Westergaard et al., 2010; DeChaine et al., 2013; Gao et al., 2015, 2017; Ebersbach et al., 2017a,b). The QTP and HHM is a biodiversity center of *Saxifraga*, harboring nearly half the species, mainly represented by the two species-rich sections *Ciliatae* Haworth and *Porphyrion* Tausch (Pan et al., 2001; Ebersbach et al., 2017a). Although immigration, recent rapid radiation, as well as lineage persistence should all contribute to the extremely high diversity and endemism of *Saxifraga* in the QTP and HHM (Ebersbach et al., 2017a), rapid geographic and adaptive radiations have proven to be disproportionally important for the QTP-HHM lineages of sects. *Ciliatae* and *Porphyrion* (Ebersbach et al., 2017b). However, nearly all of these studies have focused on the roles of diversifications or radiations on species richness of *Saxifraga* in the QTP and HHM, while case studies of intraspecific differentiations are still scarce for this species-rich *Saxifraga* region.

*Saxifraga sinomontana* J. T. Pan & Gornall is one of the common *Saxifraga* species in the QTP and HHM, inhabiting shrublands, alpine/marshy meadows and rock crevices at elevations of between 2,700 and 5,300 m a.s.l. (Pan et al., 2001). This perennial herb is extraordinarily variable in morphology, consecutively from small individuals (<5 cm) with a single flower to tall ones (more than 20 cm) with multiple flowers. This high variation probably suggests potential intraspecific diversification in this species. Phylogenetic analyses have supported a position of *S. sinomontana* in sect. *Ciliatae* subsect. *Hirculoideae* Engl. & Irmsch. (Gao et al., 2015; Tkach et al., 2015). It is characterized by pedicels with brown crisped villi as well as erect sepals with crisped villi at margins and abaxial surface (Pan et al., 2001). However, species relationships within clade of *S*. subsect. *Hirculoideae* were not well resolved in previous studies (Gao et al., 2015; Tkach et al., 2015), partly due to recent rapid radiations triggered by uplifts of the Hengduan Mountains (Ebersbach et al., 2017a,b). In fact, it seems that nearly all of the ca. 110 species in *S*. subsect. *Hirculoideae* diverged <5 Ma (Ebersbach et al., 2017a,b), or even more recently (ca. 2 Ma; Gao et al., 2015). As a member of this subsection, intraspecific differentiation of *S. sinomontana* must have occurred more recently, and probably affected by climatic oscillations during the Quaternary glaciations. Therefore, it is suitable for revealing associations between intraspecific diversification and the Quaternary climatic oscillations.

In the present study, we sequenced two chloroplast DNA (cpDNA) fragments (*trn*L-*trn*F and *rpl*16) and nuclear ribosomal DNA internal transcribed spacer (ITS) to reveal the population genetic structure and evolutionary history of *S. sinomontana*. In particular, we want to address: (1) whether intraspecific diversification has occurred in this QTP-HHM species; and (2) whether climatic oscillations during the Quaternary glaciations promoted the intraspecific diversification if it occurred.

## Materials and methods

### Sample collection

**Figure 1:**
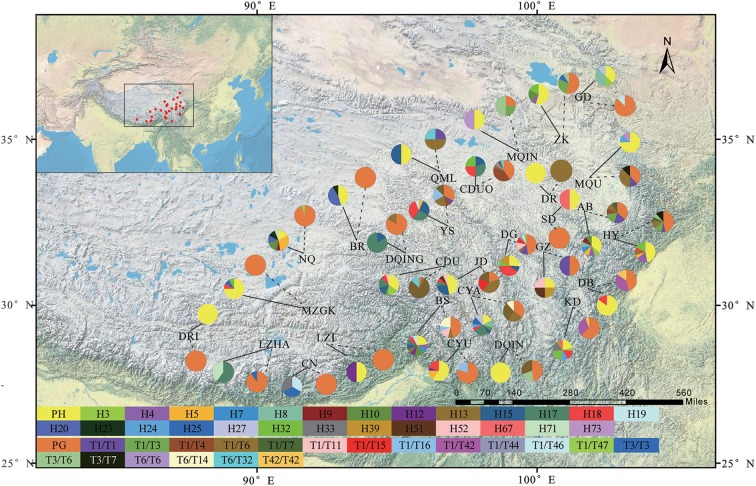
A map showing the sites of 29 sampled populations and the geographic distribution of haplotypes and genotypes in *Saxifraga sinomontana* based on the cpDNA and ITS datasets, respectively. Solid line and dashed line on the map indicating the distribution of haplotypes and genotypes of each population, respectively. Pie charts showing the proportion of haplotypes and genotypes within each population. PH, private haplotypes; PG, private genotypes. Population codes are the same as in Table 1. Names of haplotypes and genotypes are the same as in Table 2.

Fresh leaves of one to 24 individuals (according to the population size) were sampled from each population, spaced at least 5 m apart. In total, leaf materials of 350 individuals from 29 populations were collected across the extant distribution range of *S. sinomontana* (Table 1, Figure 1). Leaves were dried in silica gel. Voucher specimens of all populations are deposited in the herbarium of Northwest Institute of Plateau Biology (HNWP), Xining, Qinghai, China. *Saxifraga tangutica* Engl. was used as outgroup in the phylogenetic analyses.

**Table 1:** Population code (Pop.), sampling location, coordinates, altitude, and number of sampled individuals (*n*) of the 29 investigated populations of *Saxifraga sinomontana*.

**Pop**.	**Location**	**Latitude**	**Longitude**	**Altitude (m)**	***n***
AB	Aba, Sichuan	32°46′02″	101°40′01″	3,450	11
BS	Basu, Tibet	29°40′23″	96°43′10″	4,450	13
BR	Biru, Tibet	31°49′57″	93°33′19″	4,420	11
CYA	Chaya, Tibet	30°40′04″	97°13′35″	4,530	17
CYU	Chayu, Tibet	29°18′59″	97°01′18″	4,690	20
CDU	Changdu, Tibet	31°04′48″	96°56′59″	4,610	23
CDUO	Chengduo, Qinghai	33°12′02″	97°28′13″	4,450	20
CN	Cuona, Tibet	28°19′23″	91°55′09″	4,770	3
DR	Dari, Qinghai	33°16′48″	100°24′55″	4,190	1
DB	Danba, Sichuan	30°32′08″	101°35′27″	3,810	13
DG	Dege, Sichuan	32°03′08″	99°00′39″	4,570	23
DQIN	Deqin, Yunnan	28°23′15″	98°59′50″	4,210	23
DQING	Dingqing, Tibet	31°31′52″	95°18′40″	4,270	6
DRI	Dingri, Tibet	28°55′58″	87°26′24″	5,160	18
GZ	Ganzi, Sichuan	31°52′06″	100°16′07″	3,900	4
GD	Guide, Qinghai	36°18′20″	101°36′45″	3,490	8
HY	Hongyuan, Sichuan	32°14′18″	102°36′09″	4,300	17
JD	Jiangda, Tibet	31°20′40″	98°03′15″	4,360	15
KD	Kangding, Sichuan	30°15′17″	101°30′37″	3,550	19
LZI	Longzi, Tibet	28°37′59″	92°13′09″	5,120	2
LZHA	Luozha, Tibet	28°24′39″	90°34′31″	5,110	10
MQIN	Maqin, Qinghai	34°33′37″	99°29′35″	4,520	4
MQU	Maqu, Gansu	33°44′41″	101°52′31″	3,790	8
MZGK	Mozhugongka, Tibet	29°42′55″	92°18′05″	4,530	12
NQ	Naqu, Tibet	31°10′35″	91°45′46″	4,630	17
QML	Qumalai, Qinghai	33°58′03″	96°34′39″	4,570	4
SD	Seda, Sichuan	32°30′35″	100°23′22″	4,360	2
YS	Yushu, Qinghai	32°46′23″	97°12′17″	4,040	15
ZK	Zeku, Qinghai	35°03′07″	100°51′28″	3,660	11

### DNA extraction, PCR amplification, and sequencing

Total genomic DNA was extracted from silica-dried leaves using the modified CTAB method (Doyle and Doyle, 1987). For polymerase chain reactions (PCR), primers of “*c*” and “*f* ” for *trn*L-*trn*F (Taberlet et al., 1991), “F71” and “R1516” for *rpl*16 (Kelchner and Clark, 1997), and “18S” and “26A” for ITS (Wen and Zimmer, 1996) were employed for amplification. PCR reactions were carried out in a total volume of 50 μl containing 0.4 μl (1.5 units) of Taq polymerase, 5.0 μl of 10 × PCR buffer (with Mg^2+^), 2.0 μl of 10 mM dNTPs, 1.0 μl of 10 pM of each primer and 2.0 μl (10–20 ng) total genomic DNA. The PCR profile included an initial pretreatment of 10 min at 94°C, followed by 30 cycles of 1 min denaturation at 94°C, 50 s at primer-specific annealing temperatures, 1 min elongation at 72°C, and a final extension at 72°C for 10 min. Annealing temperatures were 49°C for *trn*L-*trn*F, 58°C for *rpl*16, and 55°C for ITS. PCR products were checked on 1% agarose gels and then purified using a CASpure PCR Purification Kit (CASarray, Shanghai, China). Purified PCR products were sequenced in both directions with the primers used for amplification on an ABI PRISM 3730xl genetic analyzer.

### Haplotype isolation

The chromatograms of each sequence were contrasted for accuracy via visual inspection with Chromas ver. 2.6.4 (available at http://www.technelysium.com.au). Primer regions were removed and DNA sequences were aligned in MEGA ver. 7.0.26 (Kumar et al., 2016) with minor subsequent adjustments. The ragged tails of the alignments were trimmed to ensure an uniform ending. All cpDNA sequences were identified to different haplotypes using DnaSp ver. 5.10 (Librado and Rozas, 2009). The two cpDNA fragments (*trn*L-*trn*F and *rpl*16) were then concatenated into a single matrix for subsequent analyses. For ITS sequences, a site was identified as heterozygous when a double peak occurred in the same position of both strands, with the weakest signal reaching at least a quarter of the strength of the strongest (Fuertes Aguilar et al., 1999; Fuertes Aguilar and Nieto Feliner, 2003). The haplotype phase for ITS sequences was reconstructed using PHASE 2.1 (Stephens et al., 2001; Stephens and Donelly, 2003) as implemented in DnaSP. Subsequent analyses of the ITS dataset were based on isolated haplotypic sequence types. All newly generated sequences in this study have been deposited in GenBank (accession no. MH432703-MH433033).

### Phylogenetic analysis

Phylogenetic relationships of the detected cpDNA/ITS haplotypes were reconstructed by means of Maximum Parsimony (MP), Maximum Likelihood (ML), and Bayesian Inference (BI). The MP analyses were conducted in PAUP 4.10b (Swofford, 2002) with all characters and transitions/transversions equally weighted. A heuristic search with 100 random-taxon-addition replicates was performed with ACCTRAN character optimization, MULPARS + TBR branch swapping, MulTrees and STEEPEST DESCENT options selected. Estimates of bootstrap support (BS) were calculated using 1,000 replicates with the above settings. jModelTest ver. 2.1.4 (Darriba et al., 2012) was used to choose the best-fit substitution models for the ML and BI analyses according to the Akaike Information Criteria (AIC). Model of GTR+I+G was selected for the cpDNA and ITS datasets. The ML analyses were conducted using RAxML ver. 8.1.21 (Stamatakis, 2014) as implemented in raxmlGUI ver. 1.5b2 (Silvestro and Michalak, 2012) with a selection of ML + rapid bootstrap and support assessment using 1,000 rapid bootstraps. The BI analyses were performed using MrBayes ver. 3.2.6 (Ronquist and Huelsenbeck, 2003; Ronquist et al., 2012) and the same substitution modes as the ML analyses. Two simultaneous Markov Chain Monte Carlo (MCMC) analyses were run for 10 million generations, sampling every 1,000 generations with the first 25% trees discarded as burn-in.

Due to limited parsimony informative sites among cpDNA/ITS haplotypes, phylogenetic reconstructions of MP, ML, and BI resulted in an almost complete lack of resolution in haplotype relationships (Supplementary Files 1, 2). We have noticed that high proportion of variable sites were represented as indels in the aligned cpDNA dataset. These indels may contain important phylogenetic information, and their involvement, to some extent, may improve phylogenetic resolution of cpDNA dataset. Indels were then coded using method described by Simmons and Ochoterena (2000) as implemented in FastGap ver. 1.2 (available at: http://www.aubot.dk/FastGap_home.htm). Phylogenetic reconstructions of indels-coded cpDNA haplotypes were repeated as described above, however, phylogenetic resolution was not yet improved (Supplementary File 1). To detect genealogical relationships among sequences with shallow genetic divergences, cpDNA/ITS haplotype networks were constructed by the program NETWORK ver. 5.0.0.3 (available at: http://www.fluxus-engineering.com), using median-joining method and MP calculation (Polzin and Daneshmand, 2003).

### Population genetic structure analysis

Average gene diversity within populations (*H*_*S*_), total gene diversity (*H*_*T*_), as well as two coefficients of population genetic differentiation, *G*_*ST*_ and *N*_*ST*_, were estimated as described by Pons and Petit (1996) using the program PERMUT (available at: http://www.pierroton.inra.fr/genetics/labo/Software/PermutSSR). To assess whether the overall population differentiation exhibited a phylogeographic structure, a comparison was made between *G*_*ST*_ and *N*_*ST*_ using a permutation test with 1,000 permutations. Significantly larger *N*_*ST*_ value compared with *G*_*ST*_ value suggests the presence of phylogeographic structure (Pons and Petit, 1996). Populations with less than three individuals were discarded from the PERMUT analysis, namely populations DR (one individual), LZI (two individuals), and SD (two individuals). Analysis of molecular variance (AMOVA) was performed using Arlequin ver. 3.5.2 (Excoffier and Lischer, 2010) to partition genetic variation within and among populations. Gene diversity (*h*) and nucleotide diversity (π) of each population were calculated using Arlequin ver. 3.5.2 (Excoffier and Lischer, 2010). We further calculated haplotype/genotype diversity at the species level as an indicator of variation by dividing the number of haplotypes/genotypes recovered by the number of individuals assayed (Oliver et al., 2006). The number of private haplotypes (*N*_*ph*_) and genotypes (*N*_*pg*_) within each population was also calculated to assess genetic diversity.

### Population demographic analysis

Mismatch distribution analysis based on the cpDNA dataset was conducted to infer the demographic history of *S. sinomontana* in the overall populations. The shape of the frequency graph of pairwise differences is expected to be multimodal in samples drawn from populations at demographic equilibrium, whereas it is usually unimodal in populations having passed through a recent demographic or range expansion with high levels of migration between neighboring demes (Slatkin and Hudson, 1991; Rogers and Harpending, 1992; Harpending et al., 1998; Ray et al., 2003; Excoffier, 2004). One thousand parametric bootstrap replicates were used to generate an expected distribution under a model of sudden demographic expansion. The sum of squared deviations (SSD) was used as a statistical test to accept or reject the hypothesis of sudden population expansion. Harpending's raggedness index (HRI, Harpending, 1994) and its significance were calculated to quantify the smoothness of the observed mismatch distribution. Neutrality tests of Tajima's *D* (Tajima, 1989) and Fu's *F*_*S*_ (Fu, 1997) based on the cpDNA dataset were also carried out to assess possible expansion. Large negative values of Tajima's *D* and Fu's *F*_*S*_ statistics should be observed under the expansion hypothesis. All of these demographic tests were implemented using Arlequin ver. 3.5.2 (Excoffier and Lischer, 2010).

### Divergence time estimation

The ITS dataset was used to estimate divergence times of detected haplotypes using BEAST ver. 1.8.4 (Drummond et al., 2012). Since ancient fossil calibrations might bias divergence time estimation toward younger ages in tip clades (Sauquet et al., 2012; Gao et al., 2017), intraspecific divergence time of *S. sinomontana* was estimated using a substitution rate of 5.03 × 10^−9^ substitutions per site per year (s/s/y) of ITS fragment as estimated by Ebersbach et al. (2017a) in Saxifragaceae. Nucleotide substitution model of GTR+I+G, a strict molecular clock and the Yule tree prior were applied. Two independent MCMC analyses were run for 10 million generations, with sampling every 1,000 generations. The maximum clade credibility tree was summarized in TreeAnnotator ver. 1.8.4 (Drummond et al., 2012) with 25% burn-in.

## Results

### Haplotype/genotype distribution and phylogenetic relationships

The alignment length of sequences of individual cpDNA fragments was 897 bp and 821 bp for *trn*L-*trn*F and *rpl*16, respectively. However, there was an adenine homoploymer at site 308 and a tyrosine homopolymer at site 355 in *trn*L-*trn*F, to ensure that variable sites were actual mutations instead of sequencing errors, nucleotides of and between homopolymers were deleted in the following analyses of this intergenic spacer. In addition, adenine and tyrosine homopolymers were also detected at sites 125 and 722, respectively, in *rpl*16, we deleted nucleotides of the two homopolymers for this fragment in the following analyses. Based on the concatenated cpDNA sequences (*trn*L-*trn*F and *rpl*16), 89 haplotypes (H1-H89) were identified (Table 2). Sequence lengths of these haplotypes varied from 1,303 to 1,420 bp, with an alignment length of 1,603 bp. Only a few haplotypes, e.g., H18 (occurred in 35 individuals from 12 populations), H17 (31 individuals from eight populations), H15 (22 individuals from nine populations), H32 (17 individuals from six populations), and H10 (10 individuals from six populations), were widespread across the extant distribution range of *S. sinomontana*. However, large amounts of haplotypes (63 out of 89) were private, i.e., restricted to single populations. Most of these exhibited extremely low level of occurrence frequency (Table 2).

**Table 2:** Haplotype and genotype composition, number of private haplotypes (*Nph*) and genotypes (*Npg*), gene diversity (*h*), as well as nucleotide diversity (π) for the 29 populations of *Saxifraga sinomontana* based on cpDNA and internal transcribed spacer (ITS) datasets.

	**cpDNA**				**ITS^a^**			
**Pop**.	**Haplotype composition**	***Nph***	***h***	**π (×100)**	**Genotype composition**	***Npg***	***h***	**π (× 100)**
AB	H1(1), H2(1), H3(1), H4(1), H5(1), H6(1), H7(1), H8(1), H9(1), H10(1), H11(1)	4	1.0000	0.0161	T1/T1(2), T1/T2(1), T1/T3(2), T1/T4(1), T1/T6(1), T1/T7(1), T1/T8(1), T1/T9(1), T5/T6(1)	4	0.7056	0.3303
BS	H4(1), H7(1), H10(2), H12(1), H13(1), H14(2), H15(1), H16(1), H17(1), H18(1), H19(1)	2	0.9744	0.0143	T1/T6(1), T1/T11(2), T1/T13(1), T1/T16(1), T6/T10(1), T6/T12(1), T6/T14(3), T6/T17(1), T11/T18(1), T15/T16(1)	6	0.8831	0.5158
BR	H20(5), H21(1), H22(4), H23(1)	2	0.7091	0.0116	T1/T19(4), T1/T20(6), T19/T21(1)	3	0.6970	0.4293
CYA	H8(2), H10(1), H15(1), H17(4), H18(1), H24(1), H25(2), H26(3), H27(2)	1	0.9118	0.0290	T1/T6(4), T3/T26(1), T4/T22(1), T6/T6(5), T6/T14(2), T6/T24(1), T6/T25(1), T22/T23(1), T26/T27(1)	6	0.7112	0.4318
CYU	H5(3), H12(1), H18(4), H28(4), H29(8)	2	0.7737	0.0121	T1/T16(4), T1/T28(3), T1/T31(1), T3/T16(2), T3/T28(1), T11/T16(6), T11/T29(2), T28/T30(1)	7	0.8256	0.2122
CDU	H3(4), H10(1), H15(3), H17(4), H18(2), H30(7), H31(1), H52(1)	2	0.8538	0.0175	T1/T6(1), T6/T6(18), T6/T32(3), T6/T81(1)	1	0.2048	0.0590
CDUO	H15(4), H17(6), H18(5), H32(5)	0	0.7842	0.0019	T1/T4(8), T1/T6(1), T1/T44(1), T1/T107(1), T6/T6(1), T6/T32(1), T6/T110(1), T6/T111(1), T6/T112(2), T6/T113(1), T7/T112(1), T108/T109(1)	7	0.8436	0.4994
CN	H19(1), H25(1), H33(1)	0	1.0000	0.0154	T3/T36(1), T33/T34(1), T35/T36(1)	3	0.9333	0.3347
DR	H34(1)	1	1.0000	0.0000	T1/T6(1)	0	1.0000	0.6974
DB	H18(2), H35(2), H36(1), H37(7), H38(1)	4	0.7051	0.0180	T1/T39(1), T1/T42(5), T3/T42(2), T37/T38(1), T40/T41(1), T42/T42(2), T42/T43(1)	5	0.7477	0.4944
DG	H15(2), H18(9), H32(2), H39(4), H40(2), H41(1), H42(1), H43(1), H44(1)	5	0.8221	0.0056	T1/T1(3), T1/T6(1), T1/T11(3), T1/T15(2), T1/T18(1), T1/T27(2), T1/T44(1), T1/T45(1), T1/T46(1), T1/T47(1), T1/T49(1), T6/T11(2), T11/T15(1), T11/T44(1), T11/T48(1), T27/T50(1)	9	0.7797	0.2958
DQIN	H45(6), H46(7), H47(8), H48(2)	4	0.7431	0.0049	T3/T6(5), T3/T51(1), T3/T54(1), T6/T6(7), T6/T51(3), T6/T52(2), T6/T53(1), T6/T54(1), T55/T56(1), T56/T57(1)	8	0.6570	0.5115
DQING	H15(1), H17(5)	0	0.3333	0.0015	T1/T6(1), T3/T59(1), T3/T61(2), T58/T59(1), T59/T60(1)	4	0.8939	0.6593
DRI	H49(18)	1	0.0000	0.0000	T62/T62(13), T62/T63(4), T63/T63(1)	3	0.2857	0.0797
GZ	H39(1), H50(1), H51(1), H52(1)	1	1.0000	0.0313	T1/T1(2), T1/T64 (1), T65/T66(1)	2	0.6429	0.2341
GD	H3(1), H8(3), H27(1), H53(2), H54(1)	2	0.8271	0.0162	T1/T46(1), T1/T67(5), T3/T67(1), T46/T46(1)	3	0.7250	0.2301
HY	H4(3), H5(1), H13(1), H18(1), H32(3), H55(3), H56(1), H57(1), H58(1), H59(1), H60(1)	6	0.9338	0.0237	T1/T1(1), T1/T3(2), T1/T6(2), T1/T7(1), T1/T65(2), T1/T69(1), T1/T71(1), T3/T6(1), T3/T7(2), T3/T30(1), T3/T70(1), T6/T68(1), T70/T72(1)	7	0.8449	0.3324
JD	H15(4), H17(1), H18(1), H51(1), H52(1), H61(1), H62(2), H63(1), H64(1), H65(1), H66(1)	6	0.9333	0.0329	T1/T6(6), T1/T15(3), T6/T6(3), T6/T22(1), T6/T29(1), T6/T73(1)	3	0.6690	0.4332
KD	H3(1), H18(3), H24(2), H32(4), H33(3), H39(1), H67(1), H68(3), H69(1)	2	0.9064	0.0104	T1/T29(1), T1/T42(6), T1/T78(2), T1/T79(2), T7/T42(1), T42/T42(1), T42/T74(1), T42/T75(1), T42/T80(1), T43/T79(1), T75/T79(1), T76/T77(1)	10	0.8165	0.4674
LZI	H12(1), H70(1)	1	1.0000	0.0153	T36/T83(1), T80/T82(1)	2	1.0000	0.8136
LZHA	H17(6), H71(4)	0	0.5333	0.0130	T1/T60(1), T1/T86(2), T1/T87(1), T3/T3(1), T3/T40(1), T30/T60(1), T30/T83(1), T30/T86(1), T84/T85(1)	8	0.9158	0.2782
MQIN	H72(1), H73(2), H74(1)	2	0.8333	0.0040	T1/T3(1), T3/T6(2), T3/T11(1)	1	0.7500	0.4134
MQU	H24(1), H73(1), H75(2), H76(1), H77(1), H78(1), H79(1)	5	0.9643	0.0313	T1/T1(1), T1/T6(3), T1/T88(2), T1/T89(1), T3/T7(1)	2	0.7333	0.3359
MZGK	H18(1), H25(1), H32(1), H80(8), H81(1)	2	0.5758	0.0090	T1/T92(1), T1/T96(1), T1/T98(1), T3/T82(1), T3/T91(1), T3/T93(1), T3/T94(1), T3/T95(1), T3/T97(1), T3/T99(1), T21/T91(1), T25/T90(1)	12	0.9094	0.5988
NQ	H5(5), H9(1), H10(3), H20(2), H23(2), H71(1), H82(1), H83(1), H84(1)	3	0.8897	0.0197	T1/T33(2), T1/T47(1), T1/T100(1), T1/T101(2), T1/T102(1), T3/T33(1), T3/T104(1), T3/T105(1), T18/T33(1), T33/T100(2), T33/T103(1), T100/T104(1), T102/T103(1), T102/T104(1)	13	0.8948	0.2787
QML	H15(2), H85(2)	1	0.6667	0.0425	T1/T1(1), T1/T6(2), T6/T32(1)	0	0.6786	0.4334
SD	H67(1), H86(1)	1	1.0000	0.0007	T76/T106(2)	1	0.6667	0.2789
YS	H5(1), H15(4), H17(4), H18(5), H19(1)	0	0.7905	0.0050	T1/T1(1), T1/T4(1), T1/T6(3), T1/T16(3), T1/T115(1), T3/T114(1), T6/T6(2), T6/T114(2), T16/T116(1)	4	0.7931	0.4704
ZK	H4(1), H10(2), H32(2), H87(2), H88(3), H89(1)	3	0.8909	0.0156	T1/T3(3), T1/T83(1), T2/T3(2), T2/T23(1), T3/T3(1), T3/T6(1), T3/T29(1), T3/T109(1)	5	0.7662	0.3001
Mean			0.8064	0.0144			0.7577	0.3948

a *Gene diversity (h) and nucleotide diversity (π) of ITS dataset were computed based on sequence types isolated by PHASE*.

*Population codes are the same as those given in Table 1*.

With respect to ITS dataset, a total of 158 ITS genotypes were discovered from the 350 individuals of *S. sinomontana* (Table 2), and their sequence length varied from 717 to 718 bp with an alignment length of 718 bp. Distribution pattern of the detected ITS genotypes was similar to those of cpDNA haplotypes, that is, a small number of widespread genotypes (e.g., T1/T6, T6/T6) vs. a high proportion (88%) of population-private genotypes. Based on the 158 different ITS genotypes, we isolated 116 haplotypes (T1-T116) using the program PHASE 2.1. The following analyses for ITS dataset were based on the isolated haplotypes.

Since some private haplotypes/genotypes were identified as singletons, i.e., represented by single individuals, which can be putatively caused by amplification and/or sequencing errors, reduplication of amplification and sequencing was carried out for such individuals to omit the extremely low error rates of methodology.

**Figure 2:**
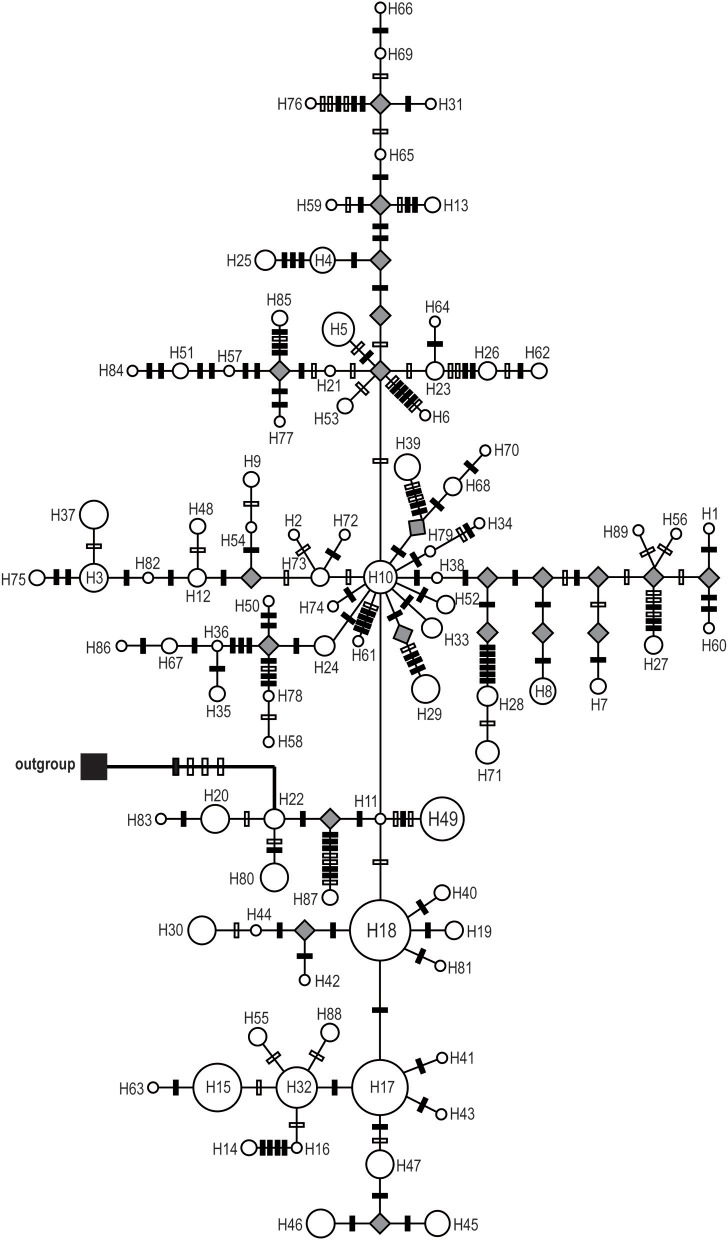
Median-joining network of the 89 cpDNA haplotypes of *Saxifraga sinomontana*. Circle size is proportional to haplotype frequencies and parallelograms represent unsampled or extinct haplotypes. Solid and open bars on the braches represent nucleotide substitutions and indels, respectively.

**Figure 3:**
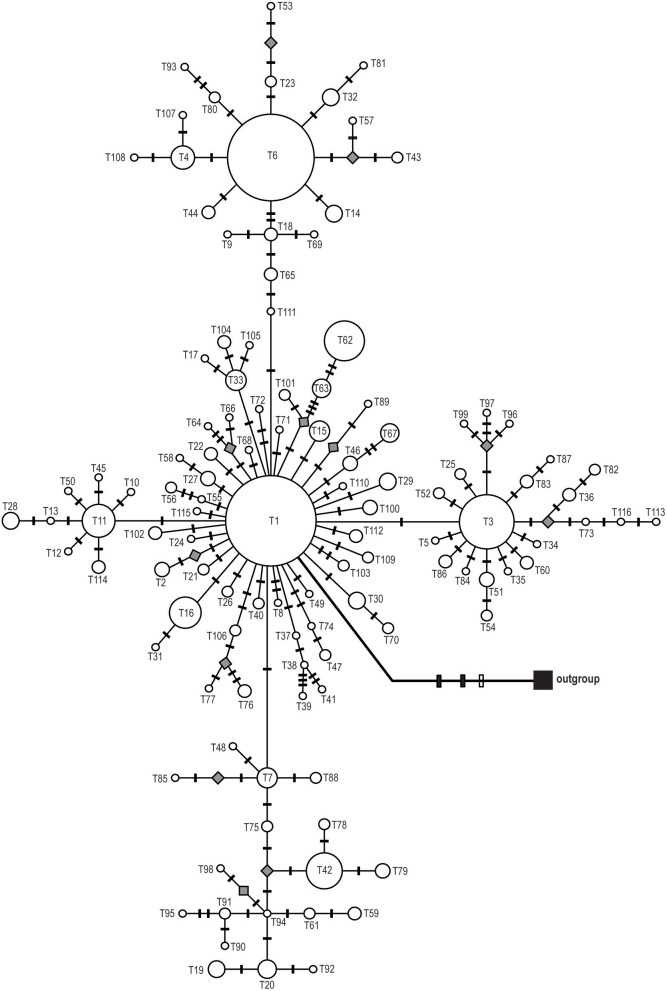
Median-joining network of the 116 ITS haplotypes of *Saxifraga sinomontana*. Circle size is proportional to haplotype frequencies and parallelograms represent missing haplotypes. Solid and open bars on the braches represent nucleotide substitutions and indels, respectively.

Phylogenetic reconstructions for both cpDNA and ITS haplotypes detected in *S. sinomontana*, by means of MP, ML, and BI, showed a “comb-like” topology. This resulted in an almost complete lack of resolution of haplotype relationships (Supplementary File 1). Median-joining networks were then conducted for these shallow-divergent cpDNA/ITS haplotypes. In both cpDNA and ITS networks, haplotypes with high distribution frequency (e.g., H10, H17, and H18 for cpDNA dataset; T1, T3, and T6 for ITS) were located in the central positions of individual networks, while population-specific haplotyes with low frequency generally occupied network tips (Figures 2, 3). In addition, the ITS haplotype network showed a star-shaped phylogeny (Figure 3). Mutation steps among neighboring cpDNA haplotypes ranged from one to nine, with the highest divergence occurring between H87 and H11/H22 by nine mutation steps (Figure 2). However, divergence among neighboring ITS haplotypes was even shallower, generally discriminated by one mutation step (Figure 3).

### Population genetic diversity and phylogeographic structure

Gene diversity (*h*) of cpDNA dataset within the 29 populations ranged from 0 to 1.0000, and nucleotide diversity (π) from 0 to 0.000425, with mean values of 0.8064 and 0.000144, respectively (Table 2). Gene diversity and nucleotide diversity based on ITS data among the 29 populations ranged from 0.2048 to 1.0000, and 0.000590 to 0.008123, with average values of 0.7577 and 0.003001, respectively (Table 2). Haplotype and genotype diversity at species level were 0.25 and 0.45, respectively. Nearly all detected populations fixed private cpDNA haplotypes and/or ITS genotypes. Both cpDNA and ITS datasets revealed high values of total genetic diversity (*H*_*T*_ = 0.978 for cpDNA, and 0.896 for ITS), as well as average within-population diversity (*H*_*S*_ = 0.784 for cpDNA, and 0.747 for ITS), suggesting a disproportionate contribution of within-population diversity to the total in *S. sinomontana*. This was confirmed by analysis of molecular variance (AMOVA), which showed that 77.94 and 60.80% of total genetic variation was found within populations based on cpDNA and ITS datasets, respectively (Table 3). However, no-significantly larger values of *N*_*ST*_ than *G*_*ST*_ were recovered for both cpDNA and ITS datasets (cpDNA: *N*_*ST*_ = 0.261, *G*_*ST*_ = 0.199, *P* > 0.05; ITS: *N*_*ST*_ = 0.354, *G*_*ST*_ = 0.166, *P* > 0.05), indicating an absence of phylogeographic structure across the extant distribution range of *S. sinomontana*.

**Table 3:** Analysis of molecular variance (AMOVA) of cpDNA haplotypes and internal transcribed spacer (ITS) sequence types for overall populations of *Saxifraga sinomontana*.

**Source of variation**	**cpDNA**					**ITS^a^**				
	***df***	***SS***	***VC***	***PV* (%)**	***F_*ST*_***	***df***	***SS***	***VC***	***PV* (%)**	***F_*ST*_***
Among populations	28	201.520	0.465	22.06		28	597.493	0.840	39.20	
Within populations	321	527.848	1.644	77.94		671	873.777	1.302	60.80	
Total	349	729.368	2.110		0.2206^*^	699	1471.271	2.142		0.3920^*^

df, degrees of freedom; SS, sum of squares; VC, variance components; PV, percentage of variation; F_ST_, fixation index;

* *P < 0.001*.

a *Analysis of molecular variance of ITS dataset was calculated based on the sequence types isolated by PHASE*.

### Population demographic analyses and divergence time

**Figure 4:**
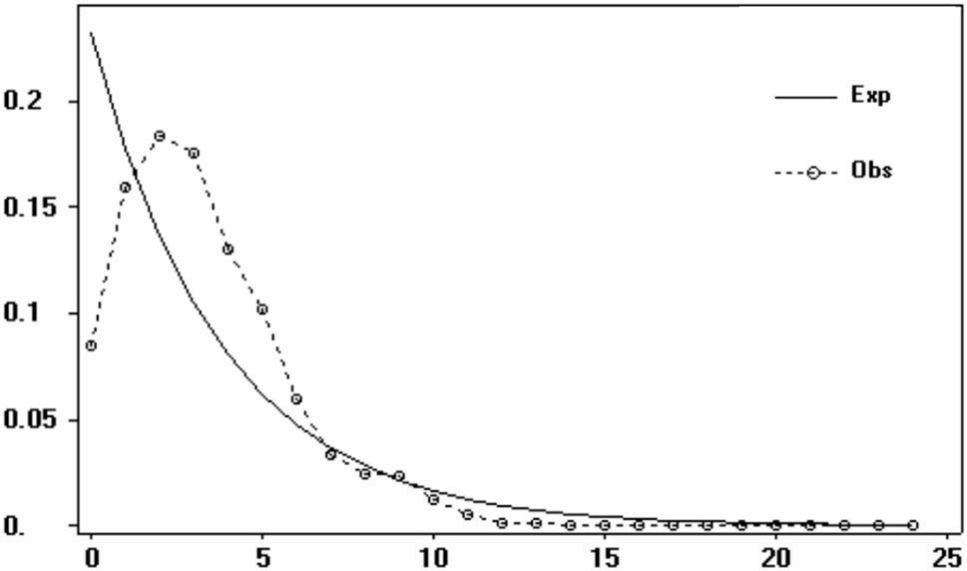
Mismatch distribution analysis of *Saxifraga sinomontana* based on overall gene pool of cpDNA dataset.

Both neutrality tests and mismatch distribution analysis based on cpDNA dataset suggested recent range or demographic expansion of *S. sinomontana*. Neutrality tests of Tajima' *D* (−2.134, *P* < 0.001) and Fu' *F*_*S*_ (−4.923, *P* < 0.001) statistics produced significantly negative values for the overall gene pool, suggesting recent expansion across the distribution range of *S. sinomontana*. This was supported by mismatch distribution analysis, in which unimodal was drawn from the overall populations (Figure 4). Besides, both indices of sum of squared deviations (SSD, 0.004, *P* > 0.05) and Harpending's raggedness index (HRI, 0.004, *P* > 0.05) were not significant, showing no deviation of observed mismatch distribution from simulation under a model of sudden demographic expansion.

**Figure 5:**
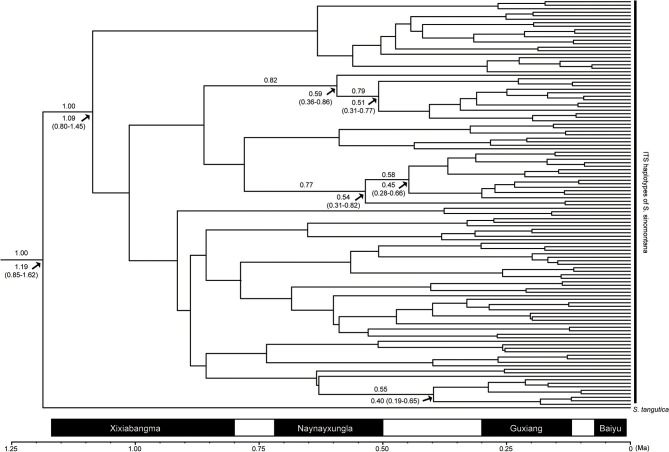
Maximum clade credibility tree of detected ITS haplotypes of *Saxifraga sinonmontana* and their divergence times estimated with the substitution rate of 5.03 × 10^−9^ s/s/y. Numbers above the branches indicate Bayesian posterior probabilities. The node age estimates are marked under branches, and their 95% highest posterior densities are shown in parentheses. Solid bars at the bottom indicate the four intensive glaciations on the Qinghai-Tibetan Plateau during the Quaternary, and open bars represent inter-glacial episodes [adapted from Zheng et al. (2002)].

The ITS dataset was employed to estimate the onset time of intraspecific divergence in *S. sinomontana*. Based on a mean substitution rate of 5.03 × 10^−9^ substitutions per site per year (s/s/y) of ITS fragments as estimated by Ebersbach et al. (2017a) in Saxifragaceae, the onset of intraspecific divergence of *S. sinomontana* was estimated to be 1.09 Ma (95% HPD = 0.80–1.45), corresponding to the Quaternary climatic oscillations (Figure 5).

## Discussion

An increasing number of plant phylogeographic studies have been conducted in the QTP and its flanking mountains (e.g., Zhang et al., 2005; Meng et al., 2007; Wang L. et al., 2009; Opgenoorth et al., 2010; Wang et al., 2010; also see review by Qiu et al., 2011). However, the aims of these studies mainly focused on the determination of glacial refugia and routes of inter-/post-glacial expansions. Rapid intraspecific differentiations of plants in this region have not been well discussed, although rapid evolutionary diversifications have been reported in an increasing number of QTP plant groups (e.g., Liu et al., 2006; Wang Y.-J. et al., 2009; Zhang et al., 2012, 2014; Favre et al., 2015, 2016; Xing and Ree, 2017; also see review by Wen et al., 2014), including the largest circum-QTP section *Ciliatae* of *Saxifraga* (Ebersbach et al., 2017b), in which *S. sinomontana* was embedded. Genetic structure of extant populations of *S. sinomontana* differs from that of infra-generic circumarctic-alpine species *S. oppositifolia* L. (Abbott and Comes, 2003) and *S. hirculus* L. (Oliver et al., 2006). However, genetic structure of *S. sinomontana* is comparable to that of sympatric *S. tangutica* (Gengji et al., 2018).

### Rapid intraspecific diversification in *S. sinomontana*

Recently, high geological heterogeneity and rapid climate oscillations have been highlighted as potential forces for species diversification in alpine plants (Simões et al., 2016; Ebersbach et al., 2017b). Rapid plant diversification could result in phylogenetic uncertainty (Richardson et al., 2001; Hughes and Eastwood, 2006; Wen et al., 2013; Ebersbach et al., 2017b) which is attributable to shallow genetic divergence among species because of limited accumulation of mutations. In addition, lineages that have experienced recent and rapid diversifications usually exhibit high level of regional endemism (Liu et al., 2006; Wang Y.-J. et al., 2009; Zhang et al., 2012, 2014; Favre et al., 2015, 2016), probably due to short migration history, geographic barriers and a lack of suitable but unoccupied habitats. Similarly, if rapid intraspecific diversifications have occurred in a plant species, large amounts but shallow divergence of haplotypes, as well as a high proportion of private haplotypes should be expected, especially for species with limited gene flow among populations. In this study, an extremely large number of cpDNA haplotypes (89) and ITS genotypes (158) are identified across the 350 individuals from 29 populations of *S. sinomontana*. The haplotype and genotype diversity (the number of haplotypes/genotypes divided by the number of individuals) at the species level of *S. sinomontana* are 0.25 and 0.45, respectively. These are higher than the values for other QTP herbaceous species, e.g., *Aconitum gymnandrum* (cpDNA haplotype diversity/ITS genotype diversity: 0.04/not available; Wang L. et al., 2009), *Pedicularis longiflora* (0.03/not available; Yang et al., 2008), *Rhodiola alsia* (0.20/0.08; Gao et al., 2012), and *Meconopsis integrifolia* (0.03/0.17; Yang et al., 2012). In fact, *S. sinomontana* seems to show the highest haplotype/genotype diversity among the studied plant species in the QTP and adjacent regions to date, although the comparison is somewhat arbitrary since different DNA fragments have been employed for different species. A high proportion of these detected haplotypes/genotypes can be described as private (71% of cpDNA haplotypes and 88% of ITS genotypes), i.e., observed in single populations. Most of these haplotypes/genotypes exhibited extremely low frequencies, with some even represented as singletons. However, limited mutations have been detected among such a large number of haplotypes, and most neighboring haplotypes are separated by limited mutational steps. This is even more apparent in the ITS haplotype network, with most differing by only a single site mutation. Limited informative mutation sites among both cpDNA and ITS haplotypes resulted in an almost complete lack of resolution of haplotype relationships as reconstructed by means of MP, ML, and BI (Supplementary Files 1, 2), indicating recent rapid intraspecific differentiation in *S. sinomontana*. Shallow divergence of detected cpDNA haplotypes can be further confirmed by low level of mean nucleotide diversity (0.000144) across assayed populations of *S. sinomontana*. In addition, it should be notable that ITS dataset revealed a relatively high level of mean nucleotide diversity (0.003948), which could be attributed to high amounts of hybridization sites. Considering haplotype richness, proportion of private haplotypes, and haplotype divergence, it is reasonable to speculate that recent intraspecific diversification occurred in this QTP-Himalayan species, *S. sinomontana*.

Global cooling and climatic oscillations, which started in the Middle Miocene and intensified continuously until the end of the Pleistocene (Miao et al., 2012; Favre et al., 2015), are considered as having played a crucial role for QTP plant diversifications (Favre et al., 2016; Xing and Ree, 2017; Ebersbach et al., 2018). Intraspecific diversification of *S. sinomontana* may also have been triggered by climatic changes but should be more recent, e.g., glacial and interglacial intervals in the Quaternary. Our molecular clock analysis estimated that the onset of diversification within *S. sinomontana* to be 1.09 Ma (95% HPD = 0.80–1.45), coinciding with the first of the four extensive Quaternary glaciations on the QTP which started ca. 1.17 Ma (Zheng et al., 2002). It should kept in mind that this time estimation was based on the mean ITS substitution rate of the large family Saxifragaceae (Ebersbach et al., 2017a), and it is probably an under-estimate since the diversification rate in clade *Ciliatae* subsect. *Hirculoideae*, which includes *S. sinomontana*, was detected to be ~2.5 times higher than the background rate (Ebersbach et al., 2017b). This means that the onset differentiation time of *S. sinomontana* may be more recent, falling well within the drastic climate oscillations of the Quaternary. It is likely that repeated developments and retreats of glaciers during Quaternary fragmented distribution range of *S. sinomontana* into isolated patches, finally facilitating rapid intraspecific radiation.

However, other factors may have also played a role to some extent in the context of rapid intraspecific diversification of *S. sinomontana*. Among them, attention should be paid to large niche breadth, which was considered to be one of the two main factors that drove rapid diversification in sect. *Ciliatae* subsect. *Hirculoideae* (Ebersbach et al., 2018). *S. sinomontana* is an extraordinarily variable species (Pan et al., 2001). Populations that inhabit humid environments at relatively low elevations, such as shrublands and alpine/marshy meadows, usually exhibit tall flowering stems (>20 cm) and bear many flowers. While populations that inhabit rock crevices and calcareous rocks near nival line exhibit loose tufts with short flowering stems (<5 cm) and solitary flower. Intermediates and reticulate variations occur between the two extreme phenotypes. The extraordinary variation in morphology of *S. sinomontana* appears to be environmentally induced (Oliver et al., 2006), and it probably indicates a large niche breadth of this species. Species with a large niche breadth usually possess wide environmental tolerance, and this enables a broad distribution range (Warren et al., 2010), which could increase the opportunity for diversification by: (1) increased chance of population separation and differentiation (Gehrke and Linder, 2011; Tanentzap et al., 2015; Matuszak et al., 2016); (2) increased ecological opportunity (Losos, 2010); and (3) low chance of extinction during climatic changes (Ebersbach et al., 2018).

Overall, the differentiation among populations of *S. sinomontana* is low as revealed by both cpDNA and ITS datasets (*G*_*ST*_ = 0.199 and 0.166, *F*_*ST*_ = 0.2206 and 0.3920 for cpDNA and ITS, respectively). One possible explanation should be efficient gene flow among populations (Brochmann et al., 2003; Oliver et al., 2006). However, a large proportion of private haplotypes/genotypes have been detected in *S. sinomontana*, and almost all populations harbor private haplotypes/genotypes, indicating limited gene flow among populations. Besides, the seeds of *S. sinomontana* have no special adaptations for dispersal. Although mechanisms of pollination and seed dispersal are not clear in *S. sinomontana*, they are probably comparable to those of *S. hirculus*, a species showing similar morphology and close phylogenetic relationships (Pan et al., 2001; Gao et al., 2015). Seeds of *S. hirculus* are mainly dispersed by dropping near the parent plants with an average dispersal distance of 13 cm (Olesen and Warncke, 1989). Furthermore, although *S. hirculus* retains partial characteristics of outcrossing, self-pollination seems to be more important for reproductive assurance in rough weather conditions because of lack of effective pollinators (Li et al., 2013). Thus, effective gene flow is not a plausible explanation for the low level of population differentiation in *S. sinomontana*. However, this pattern could be caused by recent rapid intraspecific diversification of *S. sinomontana*, which is characterized by large numbers of private haplotypes/genotypes vs. few widespread ones. High frequencies of a small number of widespread haplotypes/genotypes across populations of *S. sinomontana* could decrease population differentiation. However, a high proportion of private haplotypes/genotypes could increase within-population diversity but contribute little to among-population differentiation. This pattern could be produced by their extremely low frequency, scattered distribution and shallow divergence. This is also explicable for the detection of lack of phylogeographic structure across the distribution range of *S. sinomontana*, as also have been observed in *Potentilla glabra* (Wang L.-Y. et al., 2009), *Stellera chamaejasme* (Zhang et al., 2010), *Rhodiola alsia* (Gao et al., 2012), and *Rhodiola chrysanthemifolia* (Gao et al., 2016).

A word of caution needs to be injected here because there may be other players in this story. In particular, we do not yet know to what extent, if any, hybridization and/or introgression play a role on extant genetic structure of *S. sinomontana*. According to our field investigation, *S. sinomontana* can be locally sympatric to its close relatives *S. heleonastes* H. Smith, *S. congestiflora* Engl. & Irmsch., *S. tangutica* L., and *S. pseudohirculus* Engl., even rarely sympatric to *S. przewalskii* Engl. and *S. tibetica* Losinsk. Sometimes, individuals of *S. sinomontana* can be <10 cm apart from species mentioned above, which provides a geographic opportunity for hybridization and/or introgression. Thus, considering the revealed genetic structure of *S. sinomontana* and rapid radiation of *S*. sect. *Ciliatae* subsect. *Hirculoideae* (Gao et al., 2015; Ebersbach et al., 2017b), hybridization and/or introgression between *S. sinomontana* and its sympatric relatives cannot be ruled out. A combining study of local *S. sinomontana* populations and its close relatives in sympatry can give us a complete picture of evolution of this alpine plant complex.

### Microrefugia for *S. sinomontana* during glaciations

Phylogeographic patterns of plant species on the QTP during the Quaternary glaciations have been reviewed (Qiu et al., 2011), and their corresponding genetic structures as reflected in current populations have also been throughly discussed (Gao et al., 2016). In this study, the decline in genetic diversity from southeastern edge of the QTP to the plateau platform was not detected (e.g., Zhang et al., 2005; Meng et al., 2007), and regional differentiation centers could not be identified (e.g.,Wang L. et al., 2009; Wang L.-Y. et al., 2009; Gao et al., 2012). Instead, large numbers of private haplotypes/genotypes are scattered across the distribution range of *S. sinomontana*, and populations with high genetic diversity show an even distribution, suggesting existence of microrefugia of *S. sinomontana* on the QTP during the Quaternary glaciations. In fact, considering high heterogeneity of topography of the QTP, especially of the Hengduan Mountains (Favre et al., 2015), as well as the absence of unified glaciers (Zheng et al., 2002), it is of great opportunity to provide suitable micro-environments for cold-tolerant herbs (Gao et al., 2016), even shrubs and trees (Opgenoorth et al., 2010; Wang et al., 2010), to survive glaciations *in situ*. However, a recent expansion signal was detected across the distribution range of *S. sinomontana* by both neutrality tests and mismatch distribution analysis based on cpDNA dataset. As we discussed above, the current genetic structure of *S. sinomontana*, in conjunction with the fact of low dispersal ability of seeds, provides evidence against extensive horizontal range expansion across the distribution range of *S. sinomontana*. Thus, the detected expansion signal probably represents demographic expansion or altitudinal migration in response to repeated glacier developments and retreats, as suggested in *Rhodiola chrysanthemifolia* (Gao et al., 2016), *Potentilla glabra* (Wang L.-Y. et al., 2009), and *P. fruticose* (Shimono et al., 2010). It is likely that population of *S. sinomontana* was continuous in the QTP-HHM region before the Quaternary glaciations, with some widespread haplotypes/genotypes (e.g., H15, H17, H18 for cpDNA; T1/T6, T6/T6 for ITS) across its distribution range. The following developments and retreats of glaciers during the Quaternary may have fragmented distribution range of *S. sinomontana* into isolated patches, finally facilitating *in situ* allopatric divergence. The ancient genetic structure of *S. sinomontana* may have erased to some extent due to bottleneck effects and genetic drifts during the Quaternary glaciations, which was then replaced by current genetic pattern represented by large numbers of private haplotypes/genotypes.

## Author contributions

YL performed the experiments, analyzed the data, wrote the paper, and prepared figures and tables. Q-BG designed and conceived the study, analyzed the data and reviewed and wrote parts of the paper. Z-MG, L-KJ, and Z-HW performed parts of the experiments. S-LC conceived and designed the study.

### Conflict of interest statement

The authors declare that the research was conducted in the absence of any commercial or financial relationships that could be construed as a potential conflict of interest.
