# Supplementary material for: Rapid Intraspecific Diversification of the Alpine Species Saxifraga sinomontana (Saxifragaceae) in the Qinghai-Tibetan Plateau and Himalayas
Source: Front Genet. 2018 Sep 18;9:381. doi: 10.3389/fgene.2018.00381 (PMC6153349; doi:10.3389/fgene.2018.00381)
Supplement: Supplementary file 2 [file Table_2.DOCX]

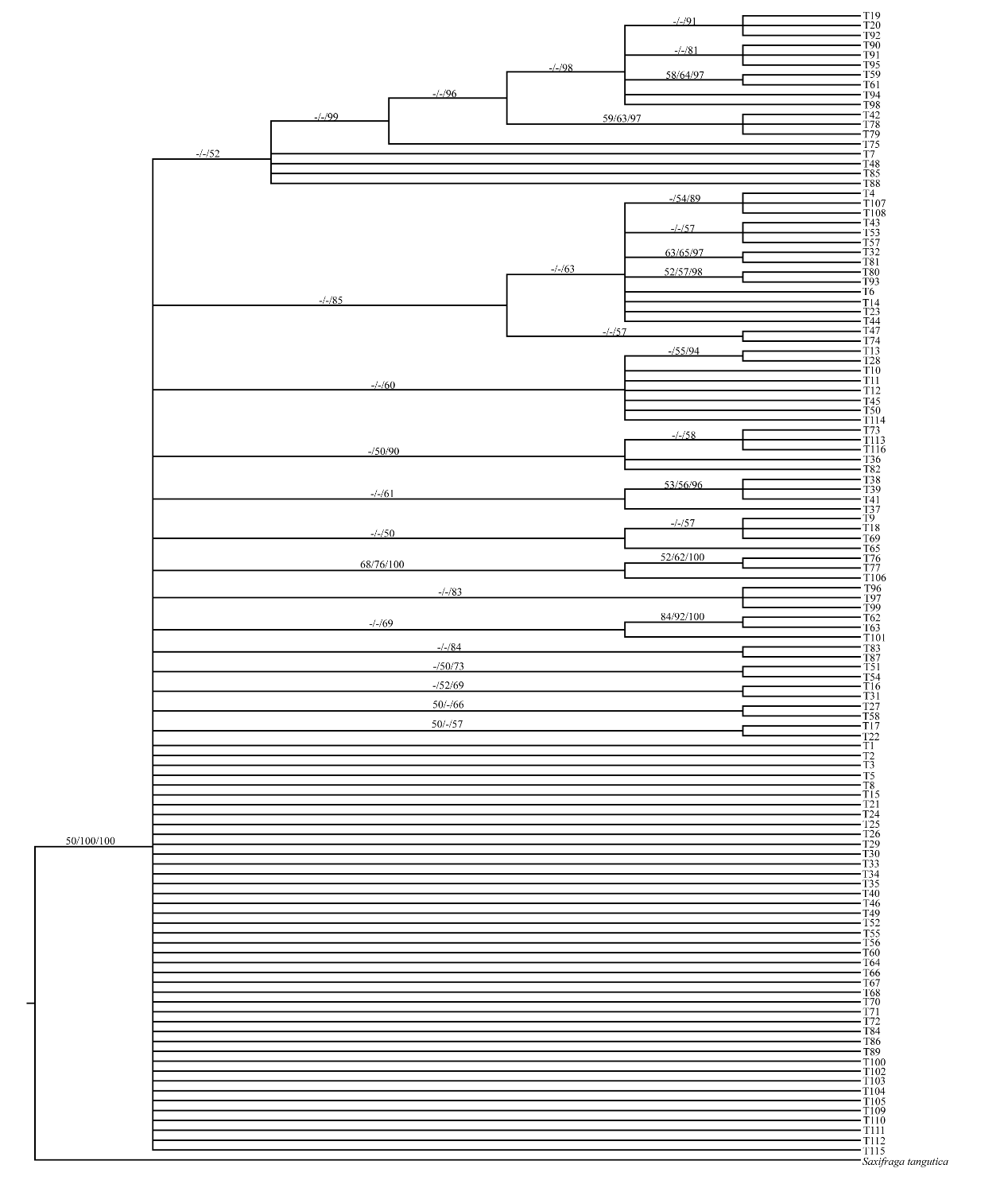


Fifty percent strict consensus trees based on ITS haplotypes of *Saxifraga sinomontana*. Numbers on the branches are bootstrap values from maximum parsimony (left) and maximum likelihood (middle) analyses and posterior probabilities (right) from Bayesian inference.
